# A Rare Case of Clear Cell Cervical Carcinoma to a Woman, 50 Years After Diethylstilbestrol Exposure for Lactation Suppression

**DOI:** 10.7759/cureus.17468

**Published:** 2021-08-26

**Authors:** Konstantinos Palaiologos, Stratos S Theofrastou, Effrosuni Gerovasileiou, Marina Flynn

**Affiliations:** 1 Obstetrics and Gynaecology, Hull University Teaching Hospitals NHS Trust, Hull, GBR; 2 Obstetrics and Gynaecology, University of Thessaly Medical School, Larissa, GRC

**Keywords:** cervical cancer, clear cell adenocarcinoma, diethylstilbestrol, cervical cancer screening, gynaecologic oncology

## Abstract

A 73-year-old lady presented with post-menopausal bleeding and a suspicious-looking endocervical polyp. She had a loop biopsy of the cervix that showed clear cell cervical carcinoma, and she was referred to our Gynaecology oncology team for further management. Following imaging for staging and an MDT discussion, she had a total abdominal radical hysterectomy that confirmed the diagnosis.

The diagnosis of clear cell adenocarcinoma of the cervix is rare, accounting for 4% of cervical carcinomas. However, it is often correlated with diethylstilbestrol (DES) exposure in utero. It is well documented that daughters of mothers who received DES during pregnancy are at a higher risk of adenocarcinomas in the genital tract. In our case, the patient had been administered DES for lactation suppression 50 years earlier. After reviewing the relevant literature, we present the case of our patient, the management of this uncommon case, and help identify possible correlation/long-term implications to patients who received DES.

## Introduction

Although cervical carcinoma is one of the most common gynaecological malignancies, the histological subtype of clear cell carcinoma is rare. It has been associated with in utero exposure to diethylstilbestrol (DES) [1]. Clear cell cervical adenocarcinoma (CCAC) is a highly invasive malignant tumour whose pathogenesis is not usually associated with human papillomavirus infection [2]. In contrast, DES is a synthetic, non-steroidal estrogen given to pregnant women to prevent losses and pregnancy-related complications from 1940 through the 1960s. The correlation between DES exposure and clear cell carcinoma of the vagina and cervix for women who have been exposed in utero is well documented by several controlled studies in current literature. This led to the foundation of the Registry for Research on Hormonal Transplacental Carcinogenesis in Boston in 1971. In the same year, DES was banned for use in pregnancy. Since then, studies investigating long-term implications of DES exposure to mothers found a modest association between DES exposure and breast cancer risk but no connection with other types of cancer [3]. There is no clear correlation yet described with cervical cancer in women who received DES and were exposed to the substance ex-utero.

This is a case of a 73-year-old woman with clear cell carcinoma of the cervix, which was found to have received DES for lactation suppression 50 years ago. It is unclear if the previous DES exposure has a relation with clear cell carcinoma of the cervix.

## Case presentation

A 73-year-old lady presented with post-menopausal bleeding and a cervical polyp. The polyp looked suspicious on colposcopy, and a loop excision of the cervix has been performed, and the sample was sent for histology. The histology results confirmed clear cell adenocarcinoma of the cervix. She had a cone biopsy in 1990 due to persistent vaginal bleeding with normal histology from her medical history. The patient has no other medical history, and she is not on any medication. We found out that she was given DES to suppress lactation 50years ago. Following the histological diagnosis, she underwent imaging with an MRI abdomen and pelvis and CT thorax for the staging of the disease and an MDT discussion with the findings.

Investigations preoperatively

Loop Diathermy Biopsy of the Cervix

Sample entirely effaced by clear cell adenocarcinoma. No normal endocervical tissue elements or transformation zone epithelium. Appearances suggest an origin from the International Federation of Gynecology and Obstetrics (FIGO) stage 1B disease. No evidence of lymphatic or vascular space permeation in the material submitted.

Staging MRI of Abdomen and Pelvis

**Figure 1:**
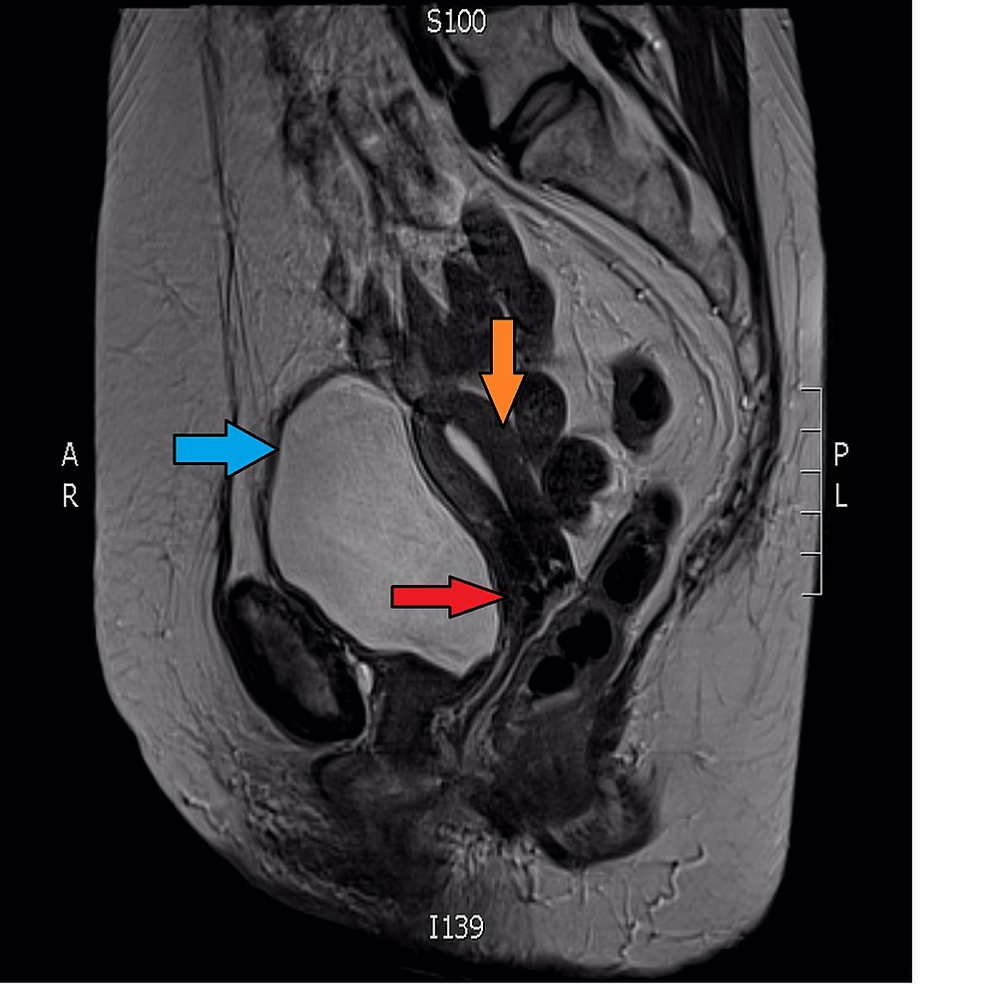
Sagittal view from MRI scan Blue arrow: Bladder; Orange arrow: Fundus of the uterus with a minimal amount of fluid in the endometrial cavity; Red arrow: Cervix

**Figure 2:**
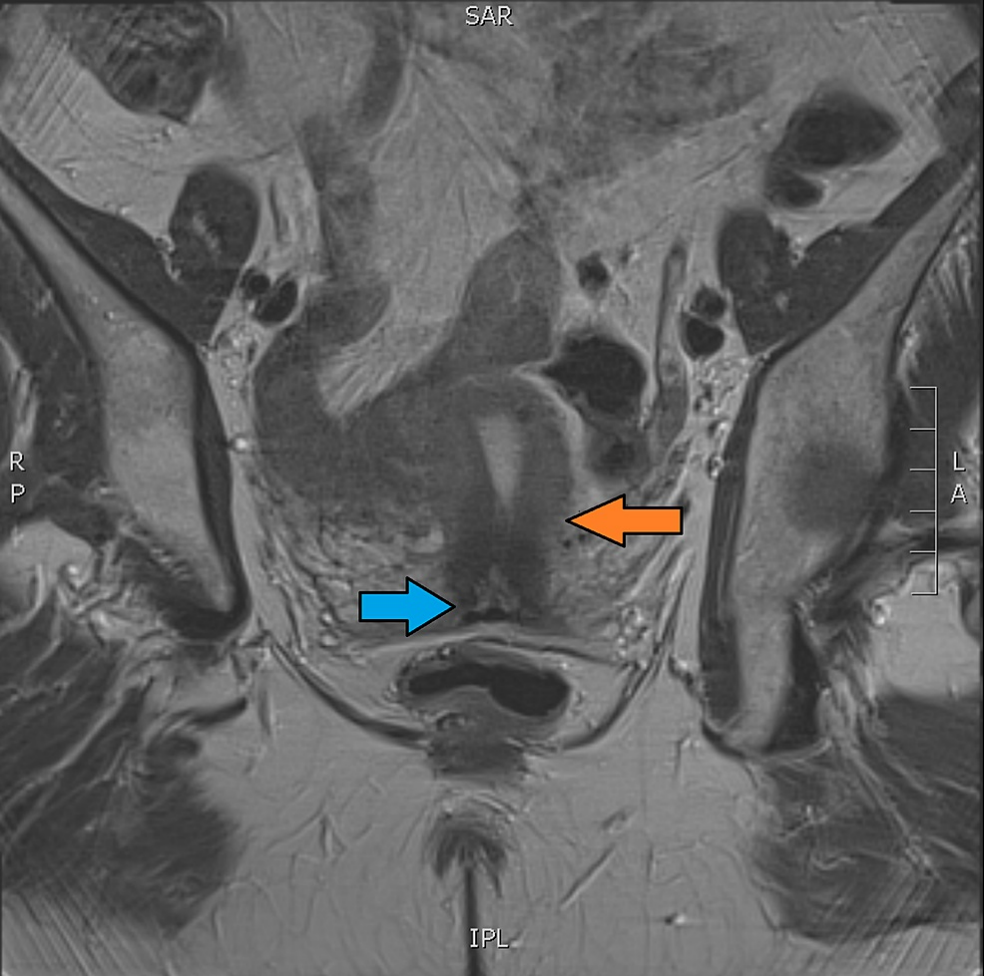
Coronal view from MRI scan Blue arrow: Cervix; Orange arrow: Isthmus of the uterus

**Figure 3:**
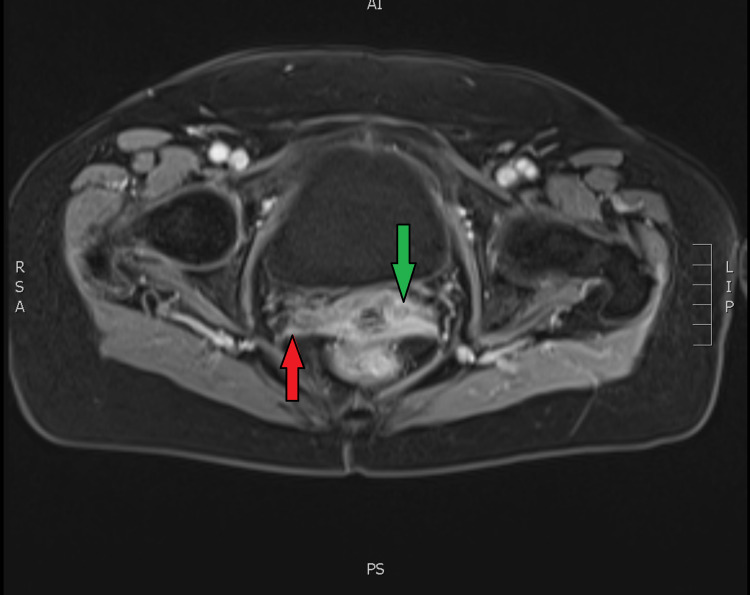
Axial view of the MRI scan Red arrow: Right parametrium; Green arrow: Axial view of the cervix

No suspicious mass or focus of restricted diffusion was seen involving the cervix. Uterus anteverted and normal in size. No focal lesion was seen. Minimal fluid in the endometrial cavity. AP dimension measures 5mm. The vagina is unremarkable. Both ovaries are normal in size and signal intensity. No suspicious lesion was seen involving the ovaries. No evidence of pelvic lymphadenopathy/peritoneal nodularity or thickening/ascites. Visualized bowel and urinary bladder unremarkable. No significant upper retroperitoneal lymphadenopathy. No subcapsular liver deposit was seen. Multiple tiny cysts-likely benign noted in the liver. Spleen, pancreas, gallbladder, adrenals, and kidneys are unremarkable. No omental/mesentery mass seen. No evidence of basal effusion. No suspicious mass or focus of restricted diffusion involving the cervix-stage 1 disease. Minimal fluid in the endometrial cavity (Figure 1-3).

CT Thorax With Contrast

No suspicious nodular lung parenchymal lesion was seen. Atelectatic changes were seen in the left lower lobe. Bilateral apical pleural thickening noted. No significant mediastinal or hilar lymphadenopathy and no evidence of pleural effusion. Conclusion: There was no definite evidence of pulmonary metastasis.

Treatment

After imaging for staging, we discussed the patient with our Gynaecological Oncology Multidisciplinary Team (MDT). She underwent total abdominal radical hysterectomy with bilateral salpingo-oophorectomy, pelvic lymphadenectomy, and omentectomy with no complications. The samples were sent for histology to confirm the diagnosis.

Outcome and follow-up

Histopathology Specimen Report

Clear Cell adenocarcinoma of the cervix, poor differentiation (grade 3). FIGO stage 1b3 due to the total size of the excised tumour 4cm, including the sample from the loop diathermy before the operation. Tumour appears on both lips of the cervix and extends upwards to reach the lower uterine segment, although no endometrial involvement/extension is seen. Endometrium: inactive, Myometrium: benign leiomyomas, Ovaries & Tubes: unremarkable, no pelvic nodes with tumour deposits, no metastatic spread to the omentum.

Follow-up

Following the histology results, the patient was discussed in the MDT meeting. Given the size of the tumour as a risk factor, adjuvant chemoradiotherapy was considered. Specifically, taxol/carbo +/- vaginal brachytherapy and surveillance were deemed reasonable options. External beam radiotherapy was not considered, given negative lymph nodes. The case was rediscussed in the MDT meeting. Given the lack of strong evidence for adjuvant treatment, and also balancing the risks and benefits and taking into account patient wishes, it was agreed by the panel for the patient to come back to the clinic to discuss her options. She finally opted for adjuvant chemotherapy. She completed six cycles of adjuvant Carboplatin and Paclitaxel chemotherapy. She is currently awaiting adjuvant brachytherapy to vaginal vault 22Gy in 4 fractions.

## Discussion

Cervical cancer is the fourth most common cancer in females. Squamous cell carcinoma of the cervix accounts for up to 75% of cervical cancer, while adenocarcinomas up to 15% [4]. The clear cell is an uncommon histological variant accounting for up to 4-9 % of the adenocarcinoma of the cervix [5,6]. Though it is a subgroup of adenocarcinoma, clinic-pathological features and prognosis are different from conventional adenocarcinoma. Association of clear cell carcinoma of cervix and vagina with in-utero exposure of DES was first published in 1971 [7]. Several articles were published during 1970-1980 regarding the increased incidence of clear cell carcinoma among the cohorts of DES exposure, and a causal association was established [8,9]. Primary clear cell carcinoma without any previous history of DES exposure is an extremely rare neoplasm. These subgroups commonly occur in elderly post-menopausal women. The median age of clear cell carcinoma non-associated with DES exposure is 53 years, and it commonly presents with irregular vaginal bleeding (80%) [8,10,11]. 

Studies regarding prognosis are conflicting; some report equivalent outcomes with conventional cervical adenocarcinoma, whereas others report a much more aggressive disease course. Important parameters for determining the prognosis of cervical clear cell carcinoma are FIGO stage, tumour size, growth pattern, nuclear atypia, mitotic activity and depth of stromal invasion.

Several studies showed a better prognosis in diethylstilbestrol-induced carcinoma than those with spontaneous clear cell carcinoma [1]. Unfavourable prognosis is related to a larger size, higher stage, high mitotic rate, positive surgical margin, parametrial involvement, and lymphovascular spread.

The treatment of clear cell carcinoma is similar to that of cervical cancer, although not well defined, and is largely based on the methods used to treat squamous cell carcinoma and non-clear cell adenocarcinoma. In the early stages, surgery is an option. Radical hysterectomy and pelvic lymphadenectomy constitute a standard surgical treatment for patients with early-stage cervical carcinoma FIGO stage IB or IIA, which results in permanent infertility in the patient. External beam radiotherapy is the standard of care for stage IIB and IIIB. The long-term effects of adjuvant radiation therapy or concurrent chemoradiation therapy may be limited for CCCUC patients with risk factors [12]. There has been an increasing focus on fertility-preserving treatment in young patients with gynaecological cancer in recent years. Vaginal radical trachelectomy and abdominal radical trachelectomy have emerged as viable options in early stages (IA-IB1) of cervical cancer without lymphatic spread in women below 45 years of age and with a strong desire to preserve fertility [13]. In more advanced stages, surgery is not recommended since it is unlikely that it will be curative. Also, advanced stages require adjuvant chemotherapy and radiotherapy, and if patients have had surgery, it is associated with a higher risk of complications [14].

In our patient as well, we did proceed with surgery and considered adjuvant chemotherapy and radiotherapy. The chemotherapy of choice is usually weekly cisplatin along with radiation. Combination chemotherapy with cisplatin and paclitaxel could also be an option, as in the case report by Thomas, where the patient had a complete response to the cisplatin and paclitaxel plus radiation [5]. One study also suggested increased activation of the EGFR-PI3K-AKT-mTOR pathway in CCAC and that inhibitor of tyrosine kinase and AKT-mTOR may be novel therapeutic targets [15].

It worths mentioning that our patient, due to her age, was not under any screening for cervical cancer. Following the current guidelines in the UK, the screening stops at 64. Moreover, the current screening program has shifted to an initial test for HPV, and if a negative HPV test, there will be no cytology (pap smear test) [16]. That might mean that we could miss patients with a clear cell adenocarcinoma of the cervix as there is no correlation with HPV. There is currently no screening for in utero DES exposure for women, although they belong in a high-risk group [17]. In research papers investigating the correlation of in utero des exposure with cervical cancer, there is a higher documented risk for CIN2+. Hence, there is a recommendation for annual pap smear tests for all those women. [18]. A study in France also suggested annual screening of cervix and vagina for these women and that it continues beyond 65-years of age and after hysterectomy with cytological examination [19]. We would need to tailor the screening program and identify those few individuals with DES exposure to aid in the early detection of cervical and vaginal adenocarcinomas [17]. Recurrence of CCAC has been reported as long as 20 years after primary therapy, emphasizing the importance of prolonging follow up.

## Conclusions

We presented a case report of a woman who developed clear cell cervical carcinoma 50 years after ex-utero exposure to DES. Clear cell carcinoma of the genital tract should prompt the clinician to take a complete medical history, including DES exposure, to the patient. The treatment of clear cell adenocarcinoma of the cervix is not well defined. We managed the case as per the MDT suggestion with a combination of primary surgical therapy and adjuvant chemotherapy plus brachytherapy due to the size of the tumour. Although CCAC is well established and studied for patients exposed to DES in utero, there are no similar cases of CCAC after ex-utero DES exposure in the current literature. There might be more implications from DES exposure to mothers that have not yet been identified in the literature. The current cervical screening policy in the UK does not have any recommendations for women at a higher risk of developing CCAC. Further studies are required to evaluate the need for more frequent and long-lasting cervical screening surveillance for these patients. However, similar studies might be difficult to be performed due to the small sample size and the barriers to identifying these subjects.
